# Assessment of the relationship between miR-499C/T (rs3746444) polymorphism and lung carcinoma in Iranian population; a case-control study

**DOI:** 10.4314/ahs.v23i3.36

**Published:** 2023-09

**Authors:** Mehdi Torabi, Mostafa Khafaei, Behnaz Jahanbin, Morteza Sadeghi

**Affiliations:** 1 Human Genetics Research Center, Baqiyatallah Medical Science University, Tehran, Iran; 2 Cancer Institute, Pathology Department, Imam Khomeini Hospital Complex, Tehran University of Medical Sciences, Tehran, Iran

**Keywords:** Lung, neoplasm, carcinoma, rs3746444, miR-499C/T, miR-499A/G, RFLP-PCR, Polymorphism, Iran

## Abstract

**Introduction:**

Lung carcinoma is characterized by uncontrollable division of respiratory system cells with detrimental and lethal consequences on human health. Critical roles of microRNAs (miR) are scientifically approved in biological and pathological pathways, such as the role of miR-499 (rs3746444) in lung carcinomas. Thus, in this case-control investigation, we aimed to assess the probable relationship between miR-499C/T variant and the occurrence of lung carcinoma in Iranian population for the first time.

**Methods:**

Genotype of miR-499 polymorphism was described by the Polymerase Chain Reaction-Restriction Fragment Length Polymorphism (PCR-RFLP) assay in patients and healthy individuals. Following definite diagnosis of lung carcinoma, the blood samples were collected, and the DNA extraction was performed by Salting-Out method. Finally, data were analysed by SPSS (v. 20) and the significant level was considered p-value<0.05.

**Results:**

Statistically, the frequency of combined genotypes of CC+CT were 83.33% and 35% and TT+CT were 100% and 92% in case and control individuals, respectively. Also, individuals with genotypes of TC (OR: 3.08, CI95%: 3.03-3.17, p<0.0001), TC+CC (OR: 0.10, CI95%: 0.05-0.23, p<0.0001), CC (OR: 0, CI95%: 0.00-0.60, p=0.0214), and TC (OR: 0.07, CI95%: 0.030.15, p<0.0001) represented statistically significant (p<0.05) differences lung carcinoma than those with TT, TT, TT+TC, and TT+CC genotypes, respectively. The frequency of miR-499C (78.5%) and miR-499T (21.5%) alleles were also statistically significantly (p<0.05) difference associated with lung carcinoma in patients than controls.

**Conclusion:**

In this study, a possible relationship among miR-499C/T polymorphism and lung carcinoma was detected in Iranian population. Since this study was conducted for the first time, thus other supplementary assessments are needed for definite conclusion.

## Introduction

Cancers are a group of pathologies associated with the irregularity of cell cycles or inhibition of physiological cell-arrest points. Thus, cell cycles are constantly repeated and proliferation is progressively and uncontrollably continued. Malignant tumors can invade surrounding tissues in a process called metastasis, leading to various clinical signs and symptoms 1. In the US, new cases of lung cancer were estimated more than 121,000 for men and 112,000 for women in 2018. Also, among 234,000 individuals with cancers, 640 cases of lung malignancies are diagnosed daily. Lung carcinoma is the 2^nd^ most common human cancer, behind prostate cancer for men and breast malignancy for women. This type of cancer is mostly diagnosed at the age of 70 years 2. Epidemiologically, there is a gradual and progressive trend in lung cancer incidence in the Iranian population over the past decades 3. Thus, investigation of the scientific bases of this type of cancer seems necessary.

Lung tumors are divided into two main categories of Non-small Cell Lung Carcinoma (NSCLC) and Small Cell Carcinoma (SCLC). More than 85% of lung tumors are NSCLC type, which includes two main categories of non-epithelial cell carcinoma (including adenocarcinoma and large cell carcinoma) and epidermoid cell carcinoma. The metastasis rate is higher in SCLC than NSCLC 4.

Single nucleotide polymorphisms (SNPs) are variable regions in the DNA sequence with rare allele frequency (1%) in the population. Like microsatellites, the SNPs are used as substitutes for genetic studies, but these SNPs contain major roles in disease incidence, unlike the microsatellites. SNPs are the most abundant variants in DNA (1/1200 bp), and their distribution is almost irregular throughout the genome 5. The miR is a small single-stranded RNA found in plants, animals, and some viruses with a critical role in RNA silencing and regulation of post-transcriptional gene expression 6. Inadequate regulation of miRNA expression occurred in most types of tumors. 50% of human miRs are located in fragile sites of DNA. Thus, the miRNAs deletion (with tumor-suppressing role) or increased level of miRNAs generation (with the oncogenic property) can be detected genetically in many types of cancers. miR-499 is a newly discovered member of miRNAs encoded by the myosin gene family. Also, the miR-499 is considered a vital molecule in the early diagnosis of common heart diseases 7.

To the best of our knowledge, no comprehensive study was conducted to assess the possible role of miR-499 mutation in lung carcinoma in the Iranian population. Thus, in this case-control study, we evaluated the probable association between miR-499C/T polymorphism and the presence of lung carcinoma in Iranian society for the first time.

## Materials and methods

### Ethical considerations

The present investigation was approved and conducted under the supervision of the Ethics Committee of Baqiyatallah University of Medical Sciences (Ethics code: IR.BMSU.REC.1399.460). All subjects enrolled in this assessment received written informed consent, filled approval forms, and donated blood samples based on personal consent. They were also allowed to leave the experiment or prevent the dissemination of information, based on their own personal opinion.

### Healthy and patient cases selection

All referred patients (from February 2020 to February 2021) to the Baqiyatallah hospital (Tehran, Iran) with the possibility of the lung carcinoma or respiratory diseases were selected for further diagnostic assessments. Since this hospital is a referral medical center in Iran and most patients are referred to this hospital from all parts of the country, thus; the final results of this project can be generalized to the whole Iranian society. Totally, 72 lung carcinoma patients (mean age of 62.82±3.2 years) and 100 healthy controls (mean age of 63.44±2.1 years) were defined as the sample's sizes based on the definite diagnosis of lung cancer. All referred patients with symptoms of dyspnea, chronic cough, hemoptysis and weight loss were collected for further evaluations. Then, by CT scan and chest X-ray assessments, the presence of pulmonary masses was examined. Finally, bronchoscopy procedure was applied for tissue sampling in order to histopathological evaluation. Thus, definite diagnosis of lung cancer was approved. 61% and 39% patients and 69% and 31% healthy individuals were males and females, respectively. Following definitive diagnosis of lung cancer, blood samples were obtained in Imam Khomeini Hospital's cancer center (Tehran, Iran) by pathologists for gene polymorphism assessment, and tissue sampling was conducted for definite lung cancer diagnosis. All biochemical and histopathological assays were also hired for definitive confirmation of lung cancer. Also, the control group was selected from the patients referred to the hospital with an initial complaint of respiratory disease. Following relevant clinical tests and radiological imaging assessments, as well as examining the history of the disease, these healthy individuals with no pathological conditions were selected as a control group.

### Materials and chemicals

Various materials, including Agarose (Cat No: 9012-36-6), Boric acid (Cat No: 10043-35-3), EDTA (Cat No: 60-00-4), and Tris- base (Cat No: 77-86-1), were prepared from Merck company (Germany). Also, Ladder 50 bp (Cat No: 32810), Self- Stain dye (Cat No: 25148), and PCR master mix (Cat No: 201289) were purchased from Sinaclone Co., and Extraction DNA Kit was obtained from Roje Co. The BCL1 restriction enzyme was supplied by New England Biolabs, Inc (Ipswich, MA, USA).

### Genomic samples collection and genotyping process

DNA content was extracted from the peripheral blood samples (2ml containing EDTA) of individuals using Salting-Out protocol 8. The purified DNA quality was assessed by electrophoresis on 3% agarose gel, and DNA concentration and purity were assessed with the Nanodrop Spectrophotometer (Thermo Fisher Scientific, Inc., Wilmington, DE, USA) at wavelengths of 260 and 280 nm. In order to assess the miR-499 polymorphism, the PCR-RFLP assay was hired. The primers below were used to amplify the miR-499 promoter region; Forward: TACAAGTACCTCGGACCCTTCAAC and Reverse: TGGAATCGCAATGCCAATTTCC (Oligo software, version 7) (Table 1) 9. 35 Cycles of PCR assay were adjusted for miR-499 amplification as the following steps; denaturation was conducted at 95oC (10min) followed by 95oC (30s). Annealing was guided at 62oC (30s), and the extension was applied at 72oC (25s). The PCR product (with 146bp) was detected using Bcll enzyme at 37oC overnight. The products of RFLP were stained using Gel Red DNA stain following electrophoresis on 3% agarose gel and visualized under ultraviolet light. Following applying the digestive effect of Bcll enzyme (at 37oC overnight) on TT position, two consequences of 120 and 26bp fragments could be detected on the gel. Also, the CC genotype was preserved in a single 146bp fragment, and the CT genotype can be distinguished in three fragments of 120, 26, and 146bp (Table 1).

**Table 1:** Forward and reverse primers of rs3746444 gene

SNP	Primers	Sequences	Tm	GC (%)	PCR product size
**rs3746444**	Forward	TACAAGTACCTCGGACCCTTCAAC	61.83	50	146 bp
	Reverse	TGGAATCGCAATGCCAATTTCC	60.42	45	

### Statistical analysis

The significant level of frequencies among the genotypes and alleles of miR-499 polymorphism were assessed using the Pearson's χ^2^ t-test (Pearson's chi-squared test) and χ^2^. Odds ratio (OR) index were analysed within the 95% confidence interval (CI95%) to determine the risk of lung carcinoma. Whole statistical analysis was applied two-sided using SPSS Software Package (v.23, IBM Corp., Armonk, NY, USA), and p-value<0.05 was considered statistically significant 10.

## Results

Significantly (p<0.05), the miR-499 polymorphism was created by C/T nucleotide transformation (miR-499C/T) with the frequency of 83.33% and 27% in healthy and cancer cases, respectively. Besides, no significant (p>0.05) alteration was detected in other genotypes of cancer patients in comparison with the healthy individuals. Also, the prevalence of miR-499C allele was found significantly (p<0.0001) higher in patients (41.66%) compared to the control individuals (21.1%). T allele (miR-499T) had significant (p<0.0001) decreased status in patients than healthy people. Thus, CT genotype was associated with the accelerated risk of lung carcinoma. The OR was 3.083 (95%CI: 3.03-3.17) for co-dominant genotype representing the 3.08-fold increased risk of lung cancer. OR index was 0.1 (95%CI: 0.05-0.23) for dominant genotype, 0 for CC (95%CI: 0.0-0.6) recessive genotype, and 0.07 (95%CI: 0.03-0.15) for over-dominant genotype. This value was also 1.38 (95%CI: 1.23-1.62) for C allele (Table 2).

**Table 2:** Distribution of miR-499C/T genotypes and the associated alleles along with odds ratio in healthy and lung cancer individuals. OR: odds ratio, CI: confidence interval

			Case N (%)	Control N (%)	OR (CI95%)	Significancy (p-value)
**miR-499C/T Genotypes**	**Codominant**	**CC**	0 (0)	8 (8)	0 (0.000-3.070)	0.5937
		**CT**	60(83.33)	27(27)	3.08308(3.03798-3.1769)	<0.0001
		**TT**	12 (16.66)	65 (65)	1	-

	**Dominant**	**TT**	12(16.66)	65(65)	1	-
		**CT+CC**	60(83.33)	35(35)	0.1077(0.05134-0.2326)	<0.0001

	**Recessive**	**TT+CT**	72(100)	92(92)	1	_-_
		**CC**	0(0)	8(8)	0(0.000-0.6068)	0.0214

	**Over dominant**	**TT+CC**	12(16.66)	73(73)	1	-
		**CT**	60(83.33)	27(27)	0.07397(0.03408-0.1560)	<0.0001

**miR-499C/T Alleles**		**T**	84(58.33)	157(78.5)	1	-
		**C**	60(41.66)	43(21.5)	1.3834(1.2377-1.6236)	<0.0001

## Discussion

Lung carcinoma originates from pulmonary or even other tissues causing malignant division of pulmonary cells and metastasis 11. It is clearly established that the lung cancer progression is directly associated with several genetic mutations. Although the molecular mechanism involved in these types of detrimental mutations is not yet fully understood, our investigation was an effort to help clarification the possible relationship among rs3746444 polymorphism and lung carcinoma. It is also approved that several genes are mutated in different cellular pathways of cancers leading to the variation of vital regulatory cellular pathways 12. Recently, various studies explained specific biomarkers involved in cancer diseases or anomalies 13. These studies showed that the lung carcinoma is a complex process associated with the abnormal expression or mutation of several vital genes needing various assessment across the world.

Probably and based on the statistically significant findings, our results indicated that the T allele is the primary allele, and the C is considered a&xa0; potential risk factor for the generation of miR-449 polymorphism. Although this finding was detected based on the statistical differences, but other complementary experiments from many parts of the world and different geographic regions are strictly needed to find the exact role of miR-449 polymorphism on occurrence of lung carcinoma. The frequency of T allele was 58.33% and 78.5% in cancer patients and control individuals, respectively. Also, the frequency of C allele was 41.66% and 21.5% in people with tumors and controls, respectively. These frequencies with a probability ratio of 1.3834 and CI95% of 1.6236-1.2377 were statistically considerable among the case and control. In the genotypes study among cancer and healthy individuals as codominant, only TT genotype was significant compared to TC, TT genotype, in Dominant it was also significant relative to TC+TT, over-dominant was CC+TT genotype compared to TC, and TT+TC genotype in recessive was statistically significant compared to CC.

Studies have shown that any change in gene expression can alter the physiological activity of the cell 14. Today, a scientific link among miR and the prognosis of various human cancers has been proven in various studies. For example, Ding and co-workers concluded that the let-7i rs10877887, miR-608 rs4919510, miR-492 rs2289030, miR-378 rs1076064, miR-423 rs6505162, miR-499 rs4919510, miR-149 rs2292832, miR-146 rs2910164, and miR-196a2 rs11614913 could serve as potential biomarkers for cancer prognosis 15. Among these, the miR-196a2 rs11614913 has a special role. Although the miR-196a2 rs11614913 polymorphism is involved in the occurrence of breast cancer, lung cancer, and colorectal cancer, but some meta-analysis study indicated that the miR-196a2 rs11614913 T variant probably contribute to decreased susceptibility to cancer. Thus, comprehensive study of miR variants and their associations with risk of cancers seems necessary for each population 16.

In 2014, a research team conducted a case-control study to investigate the possible association between polymorphisms of hsa-miR-146a (rs2910164 G> C), hsa-miR-499 (rs3746444 T> C), hsa-miRNA-196a2 (rs11614913 C> T and rs185070757 T> G), and susceptibility to the breast cancer in Iranian population 17. PCR assay was used for genotyping for miRNA SNPs. This study showed that the homozygous hsa-miR-499 rs3746444 CC can potentially increase the risk of breast cancer in dominant gene (OR: 2.42; 95 CI: 1.43-4.09; p=0.001; CC vs. TT) than recessive gene (OR: 2.48; 95%CI: 1.49-4.13; p=0.004; CC vs. TT + TC). In addition, the rs3746444 C allele increased the risk of breast cancer (OR: 1.71; 95%CI: 1.27-2.29; p=0.0004) compared to the T allele. However, the genotype distributions of rs2910164 G> C, rs11614913 C> T, and rs185070757 T> G were not statistically different between cases and controls 17. The results showed that hsa-miR-499 polymorphism is associated with a high risk of breast cancer in the Iranian population 17.

In 2013, Wu and co-workers concluded that the SNPs in miRNAs are associated with susceptibility to several human cancers./span>They evaluated the association of rs3746444 in pre-hsa-miR-499 with the risk of gastric cancer in the Chinese population 18. SNPs rs3746444 (A>G) were genotyped in 201 GC cases and 213 non-cancer individuals. There was no significant difference in rs3746444 (A>G) genotype distribution between cases and controls. In logistic regression analysis, no significant increase in GC was associated with different genotypes 19.

In 2012, a meta-analysis was performed to estimate the relationship between miR-499 polymorphism (A>G) and cancer risk. A total of 9 studies, including 6,077 cases and 7199 controls, participated in this meta-analysis. Totally, no cancer risk was significantly associated with the miR-499 G allele. In the analysis of subgroups based on ethnicity, a significant increase in the risk of cancer was observed only for Asians (dominant model: OR=1.22, 95%CI=1.02-1.46). In the subgrouping analysis (based on the cancer type), significant changes in cancer were found only for breast cancer when the miR-499 G allele was included (dominant model: OR=1.13, 95%CI=1.01-1.26). In conclusion, this meta-analysis suggested that the miR-499 polymorphism rs3746444 (A>G) is considered a low-risk factor for cancer development among Asians and also play a role in breast cancer susceptibility 20.

Many literatures have shown that MicroRNAs may be involved in cell proliferation, inflammation, oxidative stress, energy metabolism, and epithelial-mesenchymal transmission 10. Thus, MicroRNA may be involved in the development of NSCLC. In a study, patients with NSCLC patients were compared with the control groups for identification the probable relationship between miR-146a rs2910164 C/G, miR-499a rs3746444 A/G, and miR-196a rs11614913 T/C with risk factors. They found that the genotypic distribution of nucleotide polymorphisms was different in NSCLC cases and controls 21. In addition, gene-gene interaction analysis showed that the rs3746444 TC/AA could probably reduce the sensitivity to NSCLC. As a result of the total comparison, the differences among NSCLC cases and controls were not confirmed in the genetic distributions of rs2910164, rs3746444, and rs11614913 SNPs 22.

It was shown that the miR-499 could probably suppress the apoptosis process by targeting pro-apoptotic proteins of CnAα and CnAβ. A to G variability (A/G) in the miR-499 precursor can probably reduce the inhibitory effect of pri-miR-499 on CnAα/CnAβ expression representing less suppressive effect on apoptosis. Probably, this allelic changing can disrupt the RNA helix, affecting the detection of the pri- and/or pre-miR-499 and duplex miR-499 strands by RNA-binding enzymes or proteins.

In addition, the SNPs in the Seed region of miRNAs may reduce or even could lose their ability to interact with target UTR mRNAs reducing their critical role in target suppression. Or, conversely, it may alter the targets of miRNAs due to the increased desire to bind to new target sites. Thus, further studies are strictly needed to identify the precise mechanism by which the SNPs can affect miRNA expression, processing, maturation, and / or downstream effects on target genes 23.

## Conclusion

As the findings of this study showed, the miR-499C/T polymorphism was probably associated to the occurrence of lung carcinoma in Iranian population. Since this study was the first investigation evaluating the possible role of miR-499C/T polymorphism and lung carcinoma in the Iranian population, thus other complementary assessment is strictly needed to clarify the exact and real role of this polymorphism on occurrence of lung carcinoma.

## Data Availability

The datasets used and analysed for this are available from the corresponding author upon reasonable request. Ethics approval and consent to participate All assessments were conducted in accordance with ethical principles and under the supervision of the University's Ethics Committee (Ethic NO: IR.BMSU.REC.1399.460).
